# Research on the deformation law for flat rolling of a core filled tube based on the slab method

**DOI:** 10.1371/journal.pone.0237039

**Published:** 2020-08-10

**Authors:** Junlong Qi, Xianghua Liu, Haitao Gao, Xiangkun Sun

**Affiliations:** 1 State Key Laboratory of Rolling and Automation, Northeastern University, Shenyang, China; 2 Key Laboratory of Lightweight Structural Materials, Liaoning Province, Shenyang, China; Hunan University, CHINA

## Abstract

The deformation law for axisymmetric deformation during the drawing of a core filled tube (CORFT) has been studied. However, the results of such studies could not be used in the flat rolling process of the CORFT, which is a plan deformation condition. In this paper, the inner core material and outer steel tube were successively analyzed based on the slab method during the flat rolling process (plan deformation) of the CORFT, and equations for wall thickness, core density, and roll force have been developed. The theoretical results solved by the developed equations were compared with the experimental results, revealing adequate accuracy for engineering requirements. The influences of rolling parameters on the roll force and the ultimate value of the relative density of the core material were studied, and the limiting condition for a larger roll force or higher value for relative density was obtained.

## 1 Introduction

**Fig 1:**
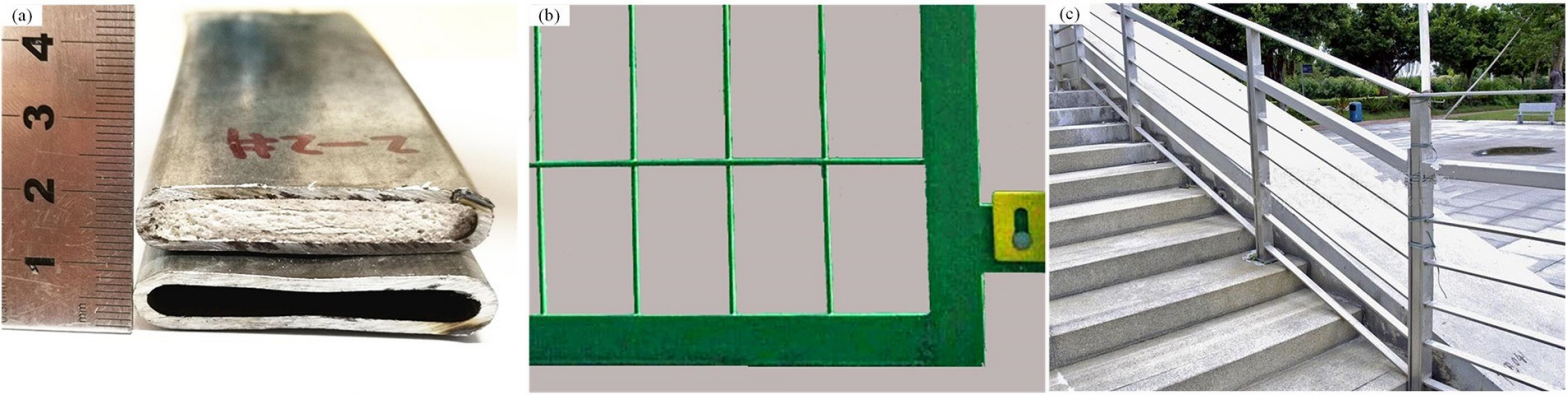
Products in which CORFTs are used. (a) Core filled tube and tube, (b) fences, and (c) stair railings.

Energy-saving [1] is an important factor for the iron and steel industry. A core filled tube (CORFT) [2] is prepared using blast furnace slag (BFS), which is typical solid waste in the iron and steel industry that has broad economic and environmental benefits. A CORFT prepared using the technology proposed in the literatures [2, 3] is shown in Fig 1(A). The CORFT is composed of Q235 steel tube and granular BFS, and it can be used as a substitute for flat steel in fences, and stair railings, as shown in (Fig 1B and 1C).

Studies of the rolling process of powder/solid composite clad rods, such as high-temperature superconducting (HTS) [4–7] and sheath materials [8, 9] have been conducted. Han et al. [10] studied the deformation law of Bi-HTS materials during the rolling process and found the critical density (the ultimate value of the powder) of the inner superconducting powder. They found that the critical density was influenced by the size and shape of the inner granule, the yield strength of the outer steel tube, and the key parameter of the ratio between the thickness and length of the deformation zone *h*/*l* but remained unchanged with different packing densities of the core material. Korzekwa et al. [11] studied the influence of process parameters on spread during the rolling of HTS materials. The parameters of concern included roll diameter, packing density, reduction rate, and the initial width of the HTS material. Pandheeradi et al. [12] adopted the finite element method to simulate the rolling process of HTS materials. Then, experiments were conducted to verify the accuracy of the simulation. Lu et al. [13] simulated the influences of initial shape on the homogeneity and density of the core material of a HTS system during the rolling process and verified the simulation by conducting experiments. Lu [14] studied the cause and influencing factors of the sausage effect (the thickness of the powder changes along with the coordinate in the length direction) for multiple HTS tapes. The rolling process was simulated using the finite element method, and the influences of roll diameter, coefficient of friction, and yield strength on the sausage effect were analyzed. Jiang et al. [15] simulated the cold rolling of a thin strip with different friction models using a three-dimensional rigid-plastic finite element method. The roll separating force, spread and forward slip for constant friction and friction variation models were compared. The friction variation in the roll bite has a significant effect on the simulation results. Cavaliere et al. [16] built a finite element model to analyze the hot rolling of steel coils and the hot mandrel rolling of a seamless steel tube.

In a previous report [17], a slab method was adopted to analyze the drawing process of the CORFT, which was an axisymmetric deformation problem. However, the previous rules cannot be used during the flat rolling process of the CORFT, which is a plan deformation problem. In this paper, first the inner core material and outer steel tube were successively analyzed based on the slab method [18–21] during the flat rolling process (plan deformation) of the CORFT, and equations for wall thickness *s*, the relative density *z* of the core material (the ratio between the core density and the granular density of the core material), and roll force *F*_d_ were developed. Then, flat rolling experiments on tubes and CORFTs were conducted, and the theoretical results solved by the developed equations were compared with the experimental results. Finally, using the theoretical equations, the influences of the rolling parameters on the roll force *F*_d_ and the ultimate value of the relative density *z*_U_ of the core material were calculated and analyzed.

## 2 Theoretical research on the flat rolling of a core filled tube

### 2.1 Parameters of the deformation zone

**Fig 2:**
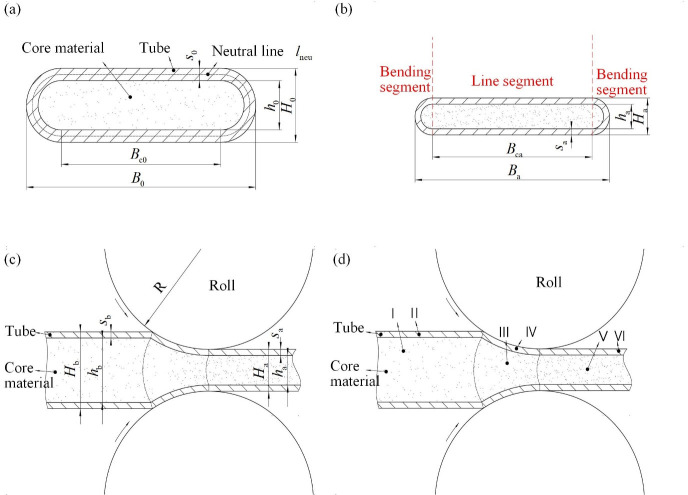
Section of the flat rolling process. (a) Cross section of preformed billet, (b) cross section at the outlet side, (c) vertical section, and (d) division of the deformation zone.

The basic parameters of the CORFT during the flat rolling process are shown in Fig 2. Fig 2(A) is the cross section of the preformed billet. Because of the low deformation, the thickness of the outer steel tube remained unchanged and the value was *s*_0_ during the pre-rolling process of the CORFT. The thickness, total width and the contact width between the rolled billet and the roll for the preformed billets are denoted as *H*_0_, *B*_0_ and *B*_c0_. As shown in (Fig 2B and 2C), the outer and inner layer of the CORFT are a steel tube and BFS, respectively. The thickness of the core material, the outer steel tube, and the total rolled billet before the flat rolling process are *h*_b_, *s*_b_ and *H*_b_, respectively, and they change into *h*_a_, *s*_a_ and *H*_a_, respectively, after the flat rolling process. The roll radius is *R* and the total width of the rolled billet and the contact width between the rolled billet and the roll are *B*_b_ and *B*_cb_ before the rolling process and become *B*_a_ and *B*_ca_ after the rolling process. The steel tube can be divided into a line segment and a bending segment, as shown in Fig 2(B). During the flat rolling process of the CORFT, the deformation zone is divided into six areas I to VI, as shown in Fig 2(D). Among the six areas, areas III and IV are deformation areas, and the other areas are the outer areas.

### 2.2 Analysis of the two stages

The flat rolling process of the CORFT can be divided into two steps: the compressive stage and the elongation stage. During the compressive stage, the holding power on the inner surface of the steel tube, which is provided by the inner core material, is not sufficient. Thus, only the outer steel tube bends; the wall thickness and the length of the steel tube remain unchanged. When the thickness of the rolled billet becomes less than the critical thickness *H*_c_, the flat rolling process transforms into the elongation stage. During this stage, the holding power is sufficient, thus the wall thickness starts to decrease, and the length of the steel tube begins to extend.

During the compressive stage, both the wall thickness and the length of the steel tube remain unchanged, or:

$$\left\{ \begin{array}{c} s_{a}=s_{0}\\ l_{\mathrm{ta}}=l_{t0} \end{array}\right.$$ (1)

During the elongation stage, neglecting the slide spread of the rolled billet, the volumetric stain (*ε*_Vb_ and *ε*_Va_) of the core material before and after the flat rolling process can be denoted as:

$$\left\{ \begin{array}{l} ‐\varepsilon_{\mathrm{Vb}}=ln\frac{h_{0}}{h_{b}∙\lambda_{0}}\\ ‐\varepsilon_{\mathrm{Va}}=ln\frac{h_{0}}{h_{a}∙\lambda_{0}∙\lambda }=‐\varepsilon_{\mathrm{Vb}}+ln\frac{h_{b}}{h_{a}∙\lambda } \end{array}\right.$$ (2)

where *ε*_Vb_ and *ε*_Va_ are the volumetric strains of the core material before and after the flat rolling process, respectively, *h*_b_ and *h*_a_ are the thicknesses of the core material before and after the flat rolling process, λ_0_ is the total accumulated elongation coefficient of the core material before the rolling pass, and λ is the elongation coefficient of the core material during the flat rolling pass.

Experiments in a previous study [17] showed that the relationship between the compressive stress *p*_y_ and the volumetric strain *ε*_V_ obeyed an exponential model, and it could be described as

$$p_{y}=A_{1}{∙exp(\frac{‐\varepsilon_{V}}{A_{2}})}^{}$$ (3)

where A_1_ and A_2_ are two constants that could be fitted from the experimental data with values of 0.390 and 0.0816, respectively.

Substituting Eq (2) into (3), the compressive stress (*p*_yb_ and *p*_ya_) of the core material before and after the flat rolling process can be denoted as:

$$\left\{ \begin{array}{l} \;p_{\mathrm{yb}}=A_{1}∙exp(\frac{‐\varepsilon_{\mathrm{Vb}}}{A_{2}})=A_{1}{\left(\frac{h_{0}}{h_{b}∙\lambda_{0}}\right)}^{\frac{1}{A_{2}}}\\ p_{\mathrm{ya}}=p_{\mathrm{yb}}∙{exp(\frac{‐\varepsilon_{\mathrm{Va}}+\varepsilon_{\mathrm{Vb}}}{A_{2}})}^{}=p_{\mathrm{yb}}∙{\left[\frac{(H_{b}‐2s_{b})∙s_{a}}{(H_{a}‐2s_{a})∙s_{b}}\right]}^{\frac{1}{A_{2}}} \end{array}\right.$$ (4)

### 2.3 Stress analysis of the inner core material

The CORFT consists of the inner core material and the outer steel tube. Thus, the force condition of the inner core material and the outer steel tube should be analyzed successively to analyze the force condition for the flat rolling process of the CORFT. To analyze the stress state of the core material, the following assumptions should be made:

1. The influence of the core material in the bending segment during the flat rolling process of the CORFT is neglected.
2. The slide spread of the outer steel tube is neglected, and the flat rolling process of the CORFT is regarded as plain deformation.
3. The coulomb friction law is used for the contact surfaces.
4. The normal stress *σ*_ix_ on a surface of the core material slab is distributed uniformly.
5. When the hydrostatic pressure *p* of the core material reaches a certain value *p*_y_, the volume of the core material shrinks, or the porosity of the core material decreases. Then,

$$\frac{\sigma_{\mathrm{ix}}+p_{\mathrm{ix}}}{2}=p_{y}$$ (5)

Or,

$$\sigma_{\mathrm{ix}}=2p_{y}{‐p}_{\mathrm{ix}}$$ (6)

where *σ*_ix_ and *p*_ix_ are the pressures of the core material in the *x* direction and are perpendicular to the contact surface between the steel tube and the core material, respectively, MPa.

**Fig 3:**
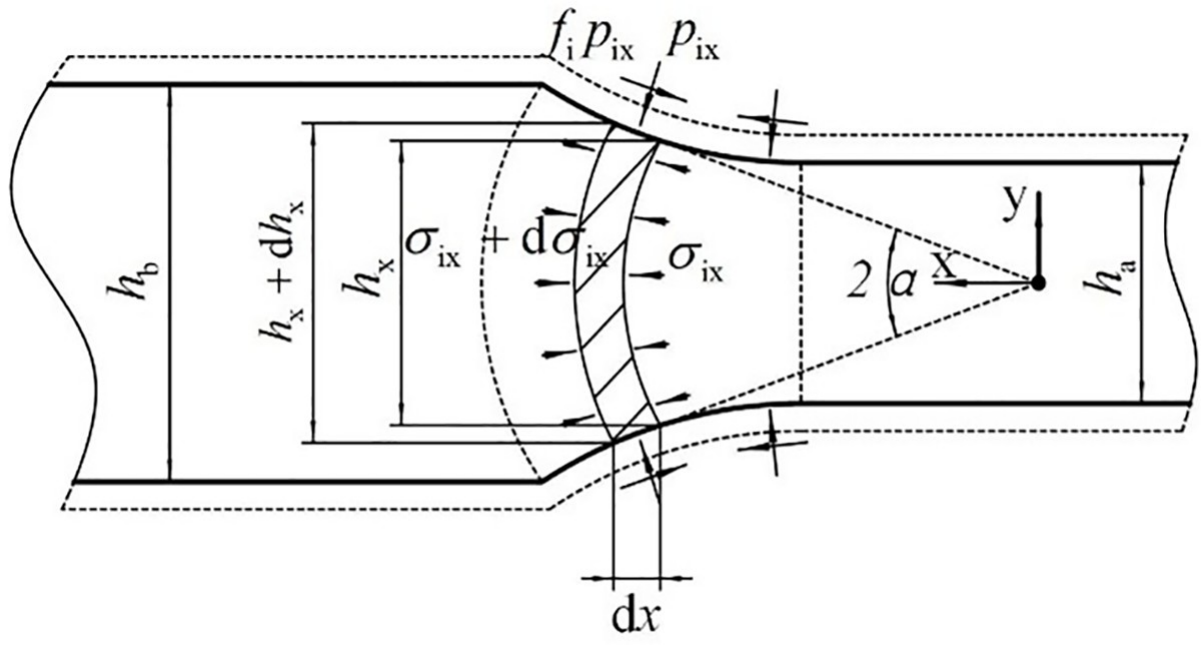
Stress analysis of the microunit of the core material.

Stress analysis is done on the microunit of the deformation zone for the core material, as shown in Fig 3.

According to the static equilibrium of the core material in the *x* direction, it can be deduced that:

$$(\sigma_{\mathrm{ix}}+d\sigma_{\mathrm{ix}})(h_{x}+dh_{x})‐\sigma_{\mathrm{ix}}h_{x}‐2p_{\mathrm{ix}}∙\frac{dx}{\cos\alpha }∙\sin\alpha \mp 2f_{i}p_{\mathrm{ix}}dx=0$$ (7)

where—and + in ∓ denote the forward and backward slide zones, respectively.

Eq (6) can be transformed into:

$$dp_{\mathrm{ix}}=‐d\sigma_{\mathrm{ix}}$$ (8)

Eqs (6), (8) and tan*α* = d*h*_x_/(2d*x*) are substituted into (7), and then,

$$\frac{dp_{\mathrm{ix}}}{dx}+\frac{\mathrm{2(}p_{\mathrm{ix}}‐p_{y})}{h_{x}}∙\frac{dh_{x}}{dx}\pm \frac{2f_{i}p_{\mathrm{ix}}}{{\mathrm{hA}}_{x}}=0$$ (9)

With a small bite angle, the contact arc can be substituted with a chord, or,

$$h_{x}=\frac{h_{b}‐h_{a}}{l}x+h_{a}$$ (10)

Or,

$$dx=\frac{l}{\Delta h}dh_{x}$$ (11)

The above equation is substituted into (9), and then,

$$\frac{dp_{\mathrm{ix}}}{(‐2\mp \frac{{2f}_{i}l}{\Delta h})p_{\mathrm{ix}}+2p_{y}}=\frac{dh_{x}}{h_{x}}$$ (12)

After integration, the equation can be denoted as follows:

$$p_{\mathrm{ix}}=C_{i}{h_{x}}^{‐2\mp \frac{{2f}_{i}l}{\Delta h}}+\frac{2p_{y}}{2\pm \frac{{2f}_{i}l}{\Delta h}}$$ (13)

At the forward slide zone, when *h*_x_ = *h*_a_, the equation can be denoted as follows:

$$p_{\mathrm{ix}}=2p_{y}‐\sigma_{\mathrm{ixa}}$$ (14)

where *σ*_ixa_ is the stress of the core material in the *x* direction at zone V, MPa.

Eq (14) is substituted into (13) while letting *ξ*_i1_ = 1-*σ*_ixa_/(2*p*_ya_) and *δ*_i1_ = 2*f*_i_*l*/Δ*h*+2, then,

$$p_{\mathrm{ix}}=\frac{{2p}_{y}}{\delta_{\mathrm{i1}}}\left[(\xi_{\mathrm{i1}}\delta_{\mathrm{i1}}‐1){\left(\frac{h_{a}}{h_{x}}\right)}^{\delta_{\mathrm{i1}}}+1\right]$$ (15)

In the backward slide zone, when *h*_x_ = *h*_b_, the equation can be denoted as follows:

$$p_{\mathrm{ix}}=2p_{y}‐\sigma_{\mathrm{ixb}}$$ (16)

where *σ*_ixb_ is the stress of the core material in the *x* direction at zone I, MPa.

Eq (16) is substituted into (13) while letting *ξ*_i2_ = 1-*σ*_ixb_/(2*p*_yb_) and *δ*_i2_ = 2*f*_i_*l*/Δ*h*-2, then,

$$p_{\mathrm{ix}}=\frac{2p_{y}}{\delta_{i2}}\left[(\xi_{i2}\delta_{i2}+1){\left(\frac{h_{x}}{h_{b}}\right)}^{\delta_{i2}}‐1\right]$$ (17)

At the demarcation plane between the forward and backward slide zones, or the neutral plane, substituting *h*_x_ = *h*_γ_ into Eqs (15) and (17) can be denoted as follows:

$$\delta_{\mathrm{i1}}(\xi_{i2}\delta_{i2}+1){h_{\gamma }}^{\delta_{\mathrm{i1}}+\delta_{i2}}‐(\delta_{\mathrm{i1}}+\delta_{i2}){h_{b}}^{\delta_{i2}}∙{h_{\gamma }}^{\delta_{\mathrm{i1}}}‐\delta_{i2}(\xi_{\mathrm{i1}}\delta_{\mathrm{i1}}‐1){h_{b}}^{\delta_{i2}}∙{h_{a}}^{\delta_{\mathrm{i1}}}=0$$ (18)

where *h*_γ_ is thickness of the core material at the neutral plan, mm and *h*_a_≤*h*_γ_≤*h*_b_.

*h*_γ_ can be determined by using a dichotomy. By integrate the parameter *p*_ix_ over the whole deformation zone, the compressive force *P*_i_ of the core material can be obtained as follows:

$$P_{i}=\frac{2p_{y}∙(B_{\mathrm{cb}}+B_{\mathrm{ca}})}{2}\times \left\{\frac{1}{\delta_{\mathrm{i1}}}\int_{0}^{x_{\gamma }}[(\xi_{\mathrm{i1}}\delta_{\mathrm{i1}}‐1){\left(\frac{h_{a}}{h_{x}}\right)}^{\delta_{\mathrm{i1}}}+1]\mathrm{dx}+\frac{1}{\delta_{i2}}\int_{x_{\gamma }}^{l}[(\xi_{i2}\delta_{i2}+1){\left(\frac{h_{x}}{h_{b}}\right)}^{\delta_{i2}}‐1]\mathrm{dx}\right\}$$ (19)

where *B*_cb_ and *B*_ca_ are the contact widths between the CORFT and the roll for the inlet and outlet side, mm, and *P*_i_ is the compressive force of the core material, MPa.

Substituting Eq (11) into the above equation can be denoted as follows:

$$P_{i}=\frac{2p_{y}∙(B_{\mathrm{cb}}+B_{\mathrm{ca}})}{2}\times \frac{l}{\Delta h}\left\{‐\frac{(\xi_{\mathrm{i1}}\delta_{\mathrm{i1}}‐1)∙h_{\gamma }}{\delta_{\mathrm{i1}}(\delta_{\mathrm{i1}}‐1)}{\left(\frac{h_{a}}{h_{\gamma }}\right)}^{\delta_{\mathrm{i1}}}‐\frac{(\xi_{i2}\delta_{i2}+1)∙h_{\gamma }}{\delta_{i2}(\delta_{i2}+1)}{\left(\frac{h_{\gamma }}{h_{b}}\right)}^{\delta_{i2}}+\frac{\xi_{\mathrm{i1}}‐1}{\delta_{\mathrm{i1}}‐1}{∙h}_{a}+\frac{\xi_{i2}‐1}{\delta_{i2}+1}{∙h}_{b}+h_{\gamma }\right\}$$ (20)

Then, the average compressive stress ${\bar{p}}_{i}$ can be solved as follows:

$${\bar{p}}_{i}=\frac{2p_{y}}{\Delta h}\left\{‐\frac{(\xi_{\mathrm{i1}}\delta_{\mathrm{i1}}‐1)∙h_{\gamma }}{\delta_{\mathrm{i1}}(\delta_{\mathrm{i1}}‐1)}{\left(\frac{h_{a}}{h_{\gamma }}\right)}^{\delta_{\mathrm{i1}}}‐\frac{(\xi_{i2}\delta_{i2}+1)∙h_{\gamma }}{\delta_{i2}(\delta_{i2}+1)}{\left(\frac{h_{\gamma }}{h_{b}}\right)}^{\delta_{i2}}+\frac{\xi_{\mathrm{i1}}‐1}{\delta_{\mathrm{i1}}‐1}{∙h}_{a}+\frac{\xi_{i2}‐1}{\delta_{i2}+1}{∙h}_{b}+(\frac{1}{\delta_{\mathrm{i1}}}+\frac{1}{\delta_{i2}})∙h_{\gamma }\right\}$$ (21)

where ${\bar{p}}_{i}$ is the average compressive stress, MPa.

### 2.4 Stress analysis for the outer steel tube

Based on the average compressive stress ${\bar{p}}_{i}$ of the core material, the stress state of the outer steel tube should be studied to analyze the stress state of the flat rolling process of the CORFT. To analyze the stress state of the outer steel tube, the following assumptions should be made:

1. The influence of the bending segment during the flat rolling process of the CORFT is neglected, and just the line segment is considered.
2. The slide spread of the outer steel tube is neglected, and the flat rolling process of the CORFT is regarded as plain deformation.
3. The coulomb friction law is used for the contact surfaces.
4. The normal stress *σ*_x_ on a same surface of the steel tube slab is distributed uniformly.
5. The approximate yield criterion of the outer steel tube can be denoted as:

$$p_{x}‐\sigma_{x}=K$$ (22)

where *σ*_x_ and *p*_x_ are pressures of the steel tube in the *x* direction and perpendicular to the contact surface between the roll and the steel tube, MPa.

**Fig 4:**
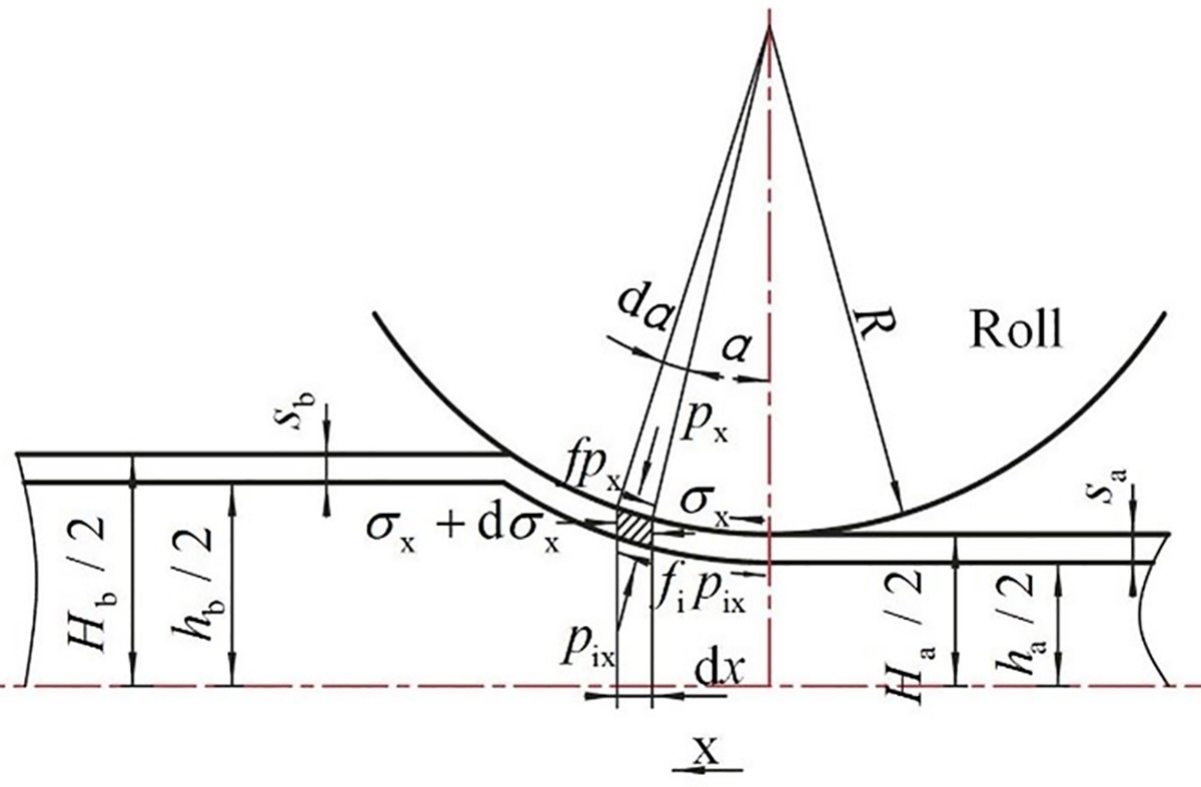
Stress analysis for the microunit of the steel tube.

As shown in Fig 4, according to the static equilibrium of the outer steel tube in the *y* direction (∑*Y* = 0), it can be denoted as follows:

$$(p_{\mathrm{ix}}‐p_{x})∙\frac{dx}{\cos\alpha }∙\cos\alpha \pm (fp_{x}‐f_{i}p_{\mathrm{ix}})∙\frac{dx}{\cos\alpha }∙\sin\alpha =0$$ (23)

Simplifying the above equation gives

$$p_{\mathrm{ix}}=\frac{1\mp f\tan\alpha }{1\mp f_{i}\tan\alpha }∙p_{x}$$ (24)

The *f*tan*α* and *f*_i_tan*α* are parameters far less than 1, so the equation can be denoted as follows:

$$p_{\mathrm{ix}}\approx p_{x}$$ (25)

Based on Eq (25), according to the static equilibrium of the outer steel tube in the *x* direction the equation can be denoted as follows:

$$(\sigma_{x}+d\sigma_{x})(s_{x}+ds_{x})‐\sigma_{x}s_{x}\mp (f‐f_{i})p_{x}∙\frac{dx}{\cos\alpha }∙\cos\alpha =0$$ (26)

where–and + in ∓ denote the forward and backward slide zones, respectively.

It can be deduced from Eq (22) that,

$$dp_{x}=d\sigma_{x}$$ (27)

Substituting Eqs (22) and (27) into (26) gives

$$\frac{dp_{x}}{dx}+\frac{p_{x}‐K}{s_{x}}\times \frac{ds_{x}}{dx}\mp \frac{f‐f_{i}}{s_{x}}p_{x}=0$$ (28)

Considering that the variation of parameter *s*_x_ is small, *s*_x_ can be assumed to change linearly in the *x* direction:

$$s_{x}=\frac{s_{b}‐s_{a}}{l}x+s_{a}$$ (29)

Or,

$$dx=\frac{l}{\Delta s}ds_{x}$$ (30)

Substituting the above Eq into (28) gives

$$\frac{dp_{x}}{ds_{x}}+\frac{p_{x}‐K}{s_{x}}\mp \frac{(f‐f_{i})∙l}{\Delta s}\times \frac{p_{x}}{s_{x}}=0$$ (31)

Integrating the above equation gives

$$p_{x}=C∙{\left(\frac{1}{s_{x}}\right)}^{1\mp \frac{(f‐f_{i})∙l}{\Delta s}}+\frac{K}{1\mp \frac{(f‐f_{i})∙l}{\Delta s}}$$ (32)

In the forward slide zone, when *s*_x_ = *s*_a_, then *p*_x_ = (1-*σ*_xa_/*K*)∙*K*. The parameter *σ*_xa_ in the above equation consists of two parts:

$$\sigma_{\mathrm{xa}}=\sigma_{f}+\sigma_{\mathrm{xai}}$$ (33)

where *σ*_f_ is the forward tension, MPa, *σ*_xai_ is the stress of the steel tube in the *x* direction deduced from the core material at the outlet side, MPa, and *σ*_xa_ is the stress of the steel tube in the *x* direction at the outlet side, MPa.

$$2s_{a}∙(\sigma_{f}+\sigma_{\mathrm{xai}})‐h_{a}{∙\sigma }_{\mathrm{ixa}}=2s_{a}∙\sigma_{f}$$ (34)

Then,

$$\sigma_{\mathrm{xai}}=\frac{h_{a}}{2s_{a}}{∙\sigma }_{\mathrm{ixa}}$$ (35)

Letting *ξ*_1_ = 1-*σ*_xa_/*K* and *δ*_1_ = (*f*-*f*_i_)∙*l*/Δ*s*-1 and substituting these two equations into (32) gives:

$$p_{x}=\frac{K}{\delta_{1}}\times \left[(\xi_{1}\delta_{1}+1)∙{\left(\frac{s_{x}}{s_{a}}\right)}^{\delta_{1}}‐1\right]$$ (36)

In the backward slide zone, when *s*_x_ = *s*_b_, then *p*_x_ = (1-*σ*_xb_/*K*)∙*K*. The parameter *σ*_xb_ in the above equation consists of two parts:

$$\sigma_{\mathrm{xb}}=\sigma_{b}+\sigma_{\mathrm{xbi}}$$ (37)

where *σ*_b_ is the backward tension, *σ*_xbi_ is the stress of the steel tube in the *x* direction deduced by the core material at the inlet side, MPa, and *σ*_xb_ is the stress of the steel tube in the *x* direction at the inlet side, MPa.

The total equilibrium of the outer end at the inlet side can be denoted as follows:

$$2s_{b}∙(\sigma_{b}+\sigma_{\mathrm{xbi}})‐h_{b}{∙\sigma }_{\mathrm{ixb}}=2s_{b}∙\sigma_{b}$$ (38)

Then,

$$\sigma_{\mathrm{xbi}}=\frac{h_{b}}{2s_{b}}{∙\sigma }_{\mathrm{ixb}}$$ (39)

Letting *ξ*_2_ = 1-*σ*_xb_/*K* and *δ*_2_ = (*f*-*f*_i_)∙*l*/Δ*s*+1, and substituting them into Eq (32) gives

$$p_{x}=\frac{K}{\delta_{2}}\times \left[(\xi_{2}\delta_{2}‐1)∙{\left(\frac{s_{b}}{s_{x}}\right)}^{\delta_{2}}+1\right]$$ (40)

Considering that the bite angle is small, the wall thickness and the thickness of the core material can be assumed to be linearly distributed along the length direction of the deformation zone, which can be denoted as follows:

$$\left\{ \begin{array}{c} s_{\gamma }=\frac{\Delta s}{l}x_{\gamma }+s_{a}\\ h_{\gamma }=\frac{\Delta h}{l}x_{\gamma }+h_{a} \end{array}\right.$$ (41)

Then,

$$s_{\gamma }=\frac{\Delta s}{\Delta h}h_{\gamma }+s_{a}‐\frac{\Delta s}{\Delta h}h_{a}$$ (42)

where *s*_γ_ is the wall thickness at the neutral plan, mm.

By integrating the parameter *p*_x_ among the whole deformation zone, the roll force *P* can be obtained as follows:

$$P=\frac{(B_{\mathrm{cb}}+B_{\mathrm{ca}})∙K}{2}\left\{\frac{1}{\delta_{1}}\int_{0}^{x_{\gamma }}[(\xi_{1}\delta_{1}+1){\left(\frac{s_{x}}{s_{a}}\right)}^{\delta_{1}}‐1]dx+\frac{1}{\delta_{2}}\int_{x_{\gamma }}^{l}[(\xi_{2}\delta_{2}‐1){\left(\frac{s_{b}}{s_{x}}\right)}^{\delta_{2}}+1]dx\right\}$$ (43)

Substituting Eq (30) into the above equation gives

$$P=\frac{(B_{\mathrm{cb}}+B_{\mathrm{ca}})∙K}{2}\times \frac{l}{\Delta s}\left\{\frac{(\xi_{1}\delta_{1}+1)∙s_{\gamma }}{\delta_{1}(\delta_{1}+1)}{\left(\frac{s_{\gamma }}{s_{a}}\right)}^{\delta_{1}}+\frac{(\xi_{2}\delta_{2}‐1)∙s_{\gamma }}{\delta_{2}(\delta_{2}‐1)}{\left(\frac{s_{b}}{s_{\gamma }}\right)}^{\delta_{2}}+\frac{1‐\xi_{1}}{\delta_{1}+1}{∙s}_{a}+\frac{1‐\xi_{2}}{\delta_{2}‐1}{∙s}_{b}‐(\frac{1}{\delta_{1}}+\frac{1}{\delta_{2}})∙s_{\gamma }\right\}$$ (44)

where *P* is the roll force, N.

Then, the average unit pressure $\bar{p}$ can be deduced as follows:

$$\bar{p}=\frac{K}{\Delta s}\left\{\frac{(\xi_{1}\delta_{1}+1)∙s_{\gamma }}{\delta_{1}(\delta_{1}+1)}{\left(\frac{s_{\gamma }}{s_{a}}\right)}^{\delta_{1}}+\frac{(\xi_{2}\delta_{2}‐1)∙s_{\gamma }}{\delta_{2}(\delta_{2}‐1)}{\left(\frac{s_{b}}{s_{\gamma }}\right)}^{\delta_{2}}+\frac{1‐\xi_{1}}{\delta_{1}+1}{∙s}_{a}+\frac{1‐\xi_{2}}{\delta_{2}‐1}{∙s}_{b}‐(\frac{1}{\delta_{1}}+\frac{1}{\delta_{2}})∙s_{\gamma }\right\}$$ (45)

where $\bar{p}$ is the average unit pressure, MPa.

### 2.5 Solving for wall thickness

During the flat rolling process of the CORFT, considering the equilibrium for the whole deformation zone in the *y* direction, the roll force *P* should be equal to the compressive force *P*_i_ of the core material, or $\bar{p}$ should be equal to ${\bar{p}}_{i}$. The thickness *s*_a_ of the outer steel tube in the equations for the parameters $\bar{p}$ and ${\bar{p}}_{i}$ are both unknown quantities and must be solved. The solution of *s*_a_ can refer to the method for solving for the roll gap using the elastoplastic curve of the mill (P-H diagram).

**Fig 5:**
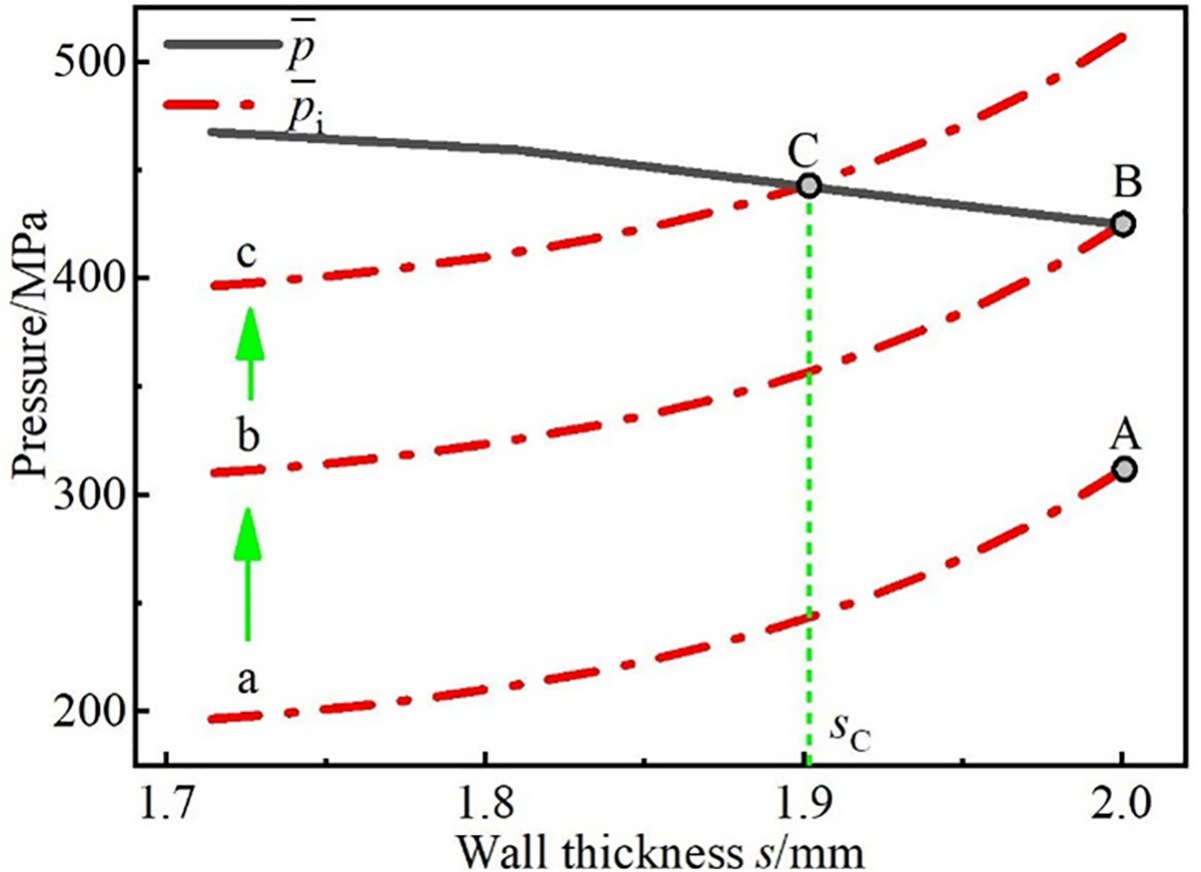
Solving for the wall thickness.

As shown in Fig 5, $\bar{p}$ and ${\bar{p}}_{i}$ are both related to the wall thickness *s*; $\bar{p}$ increases with decreasing *s*, and ${\bar{p}}_{i}$ decreases with *s*. There are three kinds of relationships between the curves $\bar{p}$ and ${\bar{p}}_{i}$ in the range of 0~*s*_b_: (a) Nonintersecting, (b) intersecting and the abscissa of the intersection point is *s*_b_, and (c) intersecting and the abscissa *s*_C_ of the intersection point is less than *s*_b_.

When the total reduction rate *e*_t_ (the reduction rate between the rolled workpiece and the preformed billet) is small, ${\bar{p}}_{i}$ can be denoted by curve a in Fig 5. In this case, the holding force on the inner surface of the steel tube provided by the core material is small because of the low core density and the wall thickness *s* of the outer steel tube changes little; thus, *s*_a_ equals *s*_b_. When *e*_t_ is large, ${\bar{p}}_{i}$ can be denoted by curve c in Fig 5. In this case, the holding force on the inner surface of the steel tube provided by the core material is sufficient because of the large core density, and the wall thickness *s* of the outer steel tube decreases, Meanwhile, after the flat rolling process of the CORFT, the wall thickness *s*_a_ equals the abscissa *s*_C_ of the intersection point, or *s*_a_ = *s*_C_. Thus, despite of the value of the total reduction rate *e*_t_, Fig 5 can be used to solve for the thickness *s*_a_.

## 3 Rolling experiments

### 3.1 Experimental procedure

Six steel tubes with dimensions of 300 mm in length, 36 mm in diameter, and 2 mm in thickness were prepared. Two tag lines (used to measure the elongation coefficient) perpendicular to the axis of each steel tube were marked to divide the steel tube into three equal parts, and the distance between the two tag lines was the initial scale length *l*_s0_. The initial outer diameters *D*_0_, initial wall thicknesses *s*_0_, initial total lengths *l*_t0_, and initial scale lengths *l*_s0_ of the steel tubes were measured. The BFS was dried in a muffle furnace at 300 degrees centigrade for 12 hours. Three portions of the dried BFS were measured to pack the three prepared steel tubes, and then three CORFT billets were obtained. The initial packing density of the billets were 0.97±0.001 g/cm^3^.

**Fig 6:**
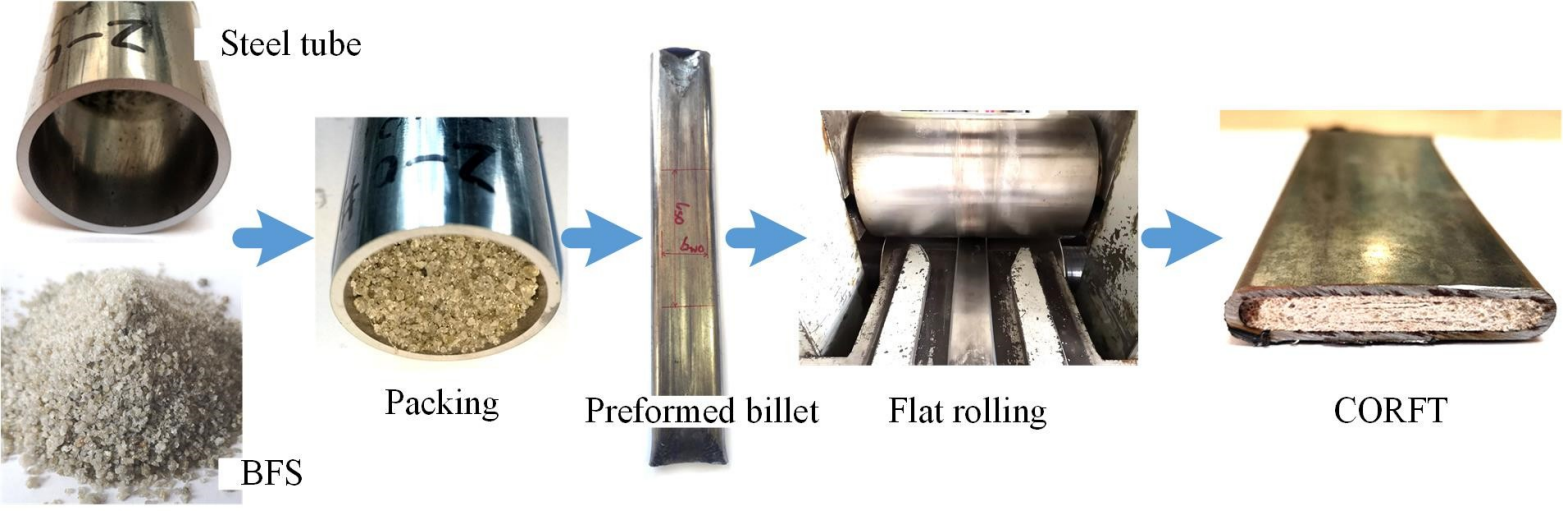
Experimental procedure of the CORFT.

When preparing CORFTs, first the oil stains on the inner and outer surfaces of the steel tubes were removed using anhydrous alcohol. Second, one end of each steel tube was flattened. After that, the prepared BFS was packed into the steel tubes, and the other side of each steel tube was flattened. Then, preformed billets (approximately 14 mm thick) were produced, and two parallel tag lines (used to measure the slide spread rate) were marked in the width direction of each preformed billet (the distance between the two tag lines was *b*_w0_). Finally, multiple passes of flat rolling were conducted, and the CORFT products 8 mm in thickness were obtained, as shown in Fig 6. Removing the packing process, the above flow can be used to prepare tubes.

The rolling mill was a 180×260 mm drag over mill. The rolling speed was adopted as 50 mm/s, and neither lubricants nor tensions were applied to the rolling process. The CORFT and the tube flat rolling experiments were repeated three times to obtain adequate accuracy.

### 3.2 Calculation of relevant parameters

During the flat rolling process of the CORFT, to obtain the relative density of the core material by using the experimental data, several hypotheses should be made, including:

(1) The wall thickness of the outer steel tube is uniform.

The bending segment elongates equally with the elongation of the line segment, because the outer steel tube is a continuum. The thickness of the bending segment decreases with elongation. Thus, the thickness of the bending segment is assumed to equal the thickness of the line segment, that is, the thickness of the outer steel tube is homogeneous and denoted as *s*_*i*_.

(2) The slide spread of the CORFR during the flat rolling process can be neglected.

During the flat rolling process of the CORFT, the total spread of the CORFT contains the slide spread, overturn spread and drum-shaped spread. When the total reduction rate (the reduction rate for the rolled billet compare with the preformed billet) is small, only the outer steel tube of the CORFT bends; thus, the slide spread of the outer steel can be neglected. When the total reduction rate is large, the parameter *B*/*l* (the ratio of total width *B* of the rolled billet and the length *l* of the contact ace) is large, the slide spread of the outer steel can also be neglected. Thus, during the flat rolling process of the CORFT, the slide spread can be neglected despite of the value of the total reduction rate.

(3) The length of the neutral line for the outer steel tube remained unchanged during the flat rolling process.

During the flat rolling process of the CORFT, the neutral line length *l*_neu_ (as shown in Fig 2(A)) of the cross section for the outer steel tube of the CORFT is not influenced by overturn spread and drum-shaped spread, and *l*_neu_ linearly increases as the slide spread increased. Thus, neglecting the slide spread, the *l*_neu_ can be assumed to remain unchanged during the flat rolling process of the CORFT.

The above hypotheses will be verified in section 4.2

The relative density *z* of the core material is the ratio of the density *ρ* and the granular density *ρ*_g_ of the core material, *z* = *ρ*/*ρ*_g_. The parameter *z* reveals the compaction rate of the core material, and determines the performance of bending resistance, flattening resistance and corrosion resisting for the CORFT.

During each flat rolling pass, the distances between the tag lines (*l*_s*i*_ and *b*_w*i*_) are measured in the length and width directions. Then, the elongation coefficient and slide spread rate after the *i*-th pass can be denoted as:

$$\left\{ \begin{array}{l} \lambda_{i}=\frac{l_{si}}{l_{si‐1}}\\ \eta_{i}=\frac{b_{wi}‐b_{\mathrm{w0}}}{b_{\mathrm{w0}}}\times 100\% \end{array}\right.$$ (46)

where λ_*i*_ and *η*_i_ are the elongation coefficient and slide spread rate of the *i*-th pass, *l*_s*i*-*1*_ and *l*_s*i*_ are the scale lengths in the length direction before and after the *i*-th pass, mm, while *b*_w*i*_ and *b*_w0_ are the scale lengths in the width direction after the *i*-th pass and for the preformed billet, mm.

Considering the mass conservation of the outer steel tube, the following equation can be obtained:

$$\rho_{s}∙s_{0}∙b_{w0}∙l_{s0}=\rho_{s}∙s_{i}∙b_{\mathrm{w0}}∙l_{s0}∙\prod_{j=1}^{i}\lambda_{i}$$ (47)

where $\prod_{j=1}^{i}\lambda_{i}=\lambda_{1}\times \lambda_{2}\times \cdots \times \lambda_{i},$ *ρ*_s_ is the density of the steel tube, g/cm^3^, *l*_s0_ and *b*_w0_ are the distances between the parallel flag lines, respectively, in the length and width directions, mm, *s*_0_ is the initial thickness of the steel tube, mm, *l*_0_ is the initial length of the steel tube, mm, and *s*_*i*_ is the wall thickness after the *i*-th pass.

Then, *s*_*i*_ can be deduced as follows:

$$s_{i}=\frac{s_{0}}{\prod_{j=1}^{i}\lambda_{i}}$$ (48)

During the flat rolling process of the CORFT, the length of neutral line can be assumed to remain unchanged. Then,

$$\pi ∙(D_{0}‐s_{0})=\pi ∙(H_{ai}‐s_{i})+2B_{ci}$$ (49)

where *H*_a*i*_ is the thickness of the rolled billet after the *i*-th pass, and *B*_c*i*_ is the contact width between the rolled billet and the roll after the *i*-th pass.

*B*_c*i*_ can be deduced from the above equation as shown:

$$B_{ci}=\frac{\pi ∙(D_{0}‐H_{ai}‐s_{0}+s_{i})}{2}$$ (50)

The section areas for the core material of the rolled billet before and after deformation (*F*_0_ and *F*_*i*_) can be obtained as follows:

$$\left\{ \begin{array}{l} F_{0}=\frac{\pi }{4}∙{\left(D_{0}‐2s_{0}\right)}^{2}\\ F_{i}=\frac{\pi }{4}∙{\left(H_{ai}‐2s_{i}\right)}^{2}+B_{ci}∙(H_{ai}‐2s_{i}) \end{array}\right.$$ (51)

where *F*_0_ and *F*_*i*_ are the section areas for the core material of the preformed billet and the rolled billet after the *i*-th pass, mm^2^.

Considering the mass conservation of the core material, it can be deduced that:

$$z_{0}∙\rho_{g}∙F_{0}=z_{i}∙\rho_{g}∙F_{i}∙\prod_{j=1}^{i}\lambda_{i}$$ (52)

where *z*_0_ and *z*_*i*_ are the initial relative density and the relative density after the *i*-th pass of the core material, and *ρ*_g_ is the density of the BFS particles, g/cm^3^.

By substituting Eq (51) into (52), *z*_*i*_ can be obtained:

$$z_{i}=\frac{\frac{\pi }{4}∙{\left(D_{0}‐2s_{0}\right)}^{2}}{[\frac{\pi }{4}∙{\left(H_{ai}‐2s_{i}\right)}^{2}+B_{ci}∙(H_{ai}‐2s_{i})]∙\prod_{j=1}^{i}\lambda_{i}}\times z_{0}$$ (53)

## 4 Results and discussion

### 4.1 Comparison of the CORFT and the tube experimental results

**Fig 7:**
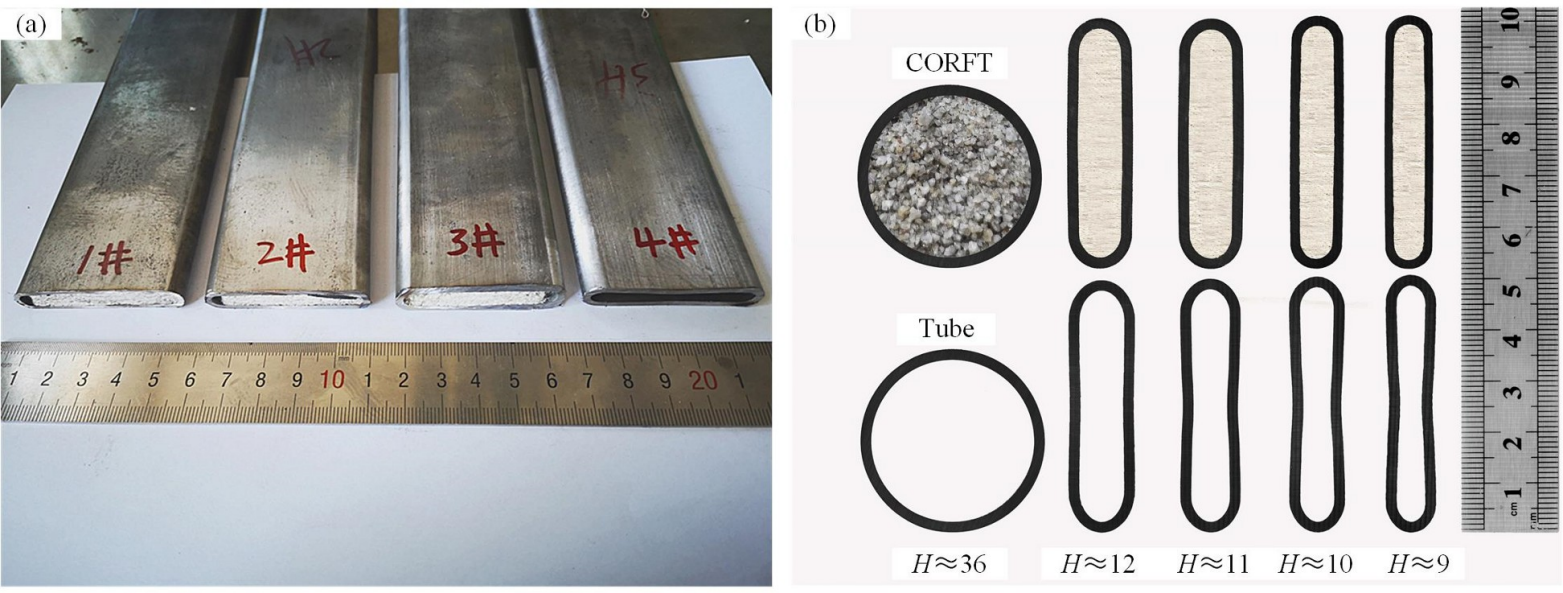
Pictures of the CORFTs and the tube. (a) Products, and (b) scanning image of the section.

Multiple passes of flat rolling experiments on the CORFTs and the tubes were conducted, and the products of the CORFT and the tube were obtained and are shown in Fig 7(A). Sections of the rolled billets were cut out with total thicknesses (*H*) of approximately 36, 12, 11, 10 and 9 mm. The scan images of the sections of the CORFTs and the tubes are shown in Fig 7(B). As shown in Fig 7(B), the wall thickness of the CORFTs decreased and the wall thickness of the tubes remained unchanged with the decrease in *H*. For the CORFTs, the normal pressure *p*_ix_ on the inner surfaces of the steel tubes provided by the core material became sufficient to promote the thinning of the steel tubes when *H* decreased to a certain value. In this case, the wall thickness decreased with the increase of the flat rolling passes. For the tubes, the normal pressure on the inner surfaces of the steel tubes equaled zero because the inner surfaces of the steel tubes were free surfaces. Thus, the outer steel tubes bent only with the increase of the flat rolling passes, and the wall thickness remained unchanged.

The original data of the workpieces after the flat rolling process of the CORFT are shown in Table 1. The table includes thickness *H*_*i*_, total width *B*_*i*_, and the distances between the flag lines in the length direction *l*_s*i*_ and the width direction *b*_w*i*_.

**Table 1:** Original data of the flat rolling experiments of the CORFT (mm).

Thickness *H*_*i*_	Total width *B*_*i*_	Distance between flag lines in length direction *l*_si_	Distance between flag lines in width direction *b*_wi_
14.01	47.60	100.04	31.15
12.04	48.74	101.79	30.98
11.03	49.14	106.580	31.30
9.98	49.53	116.630	31.60
8.96	49.90	128.750	31.91

**Fig 8:**
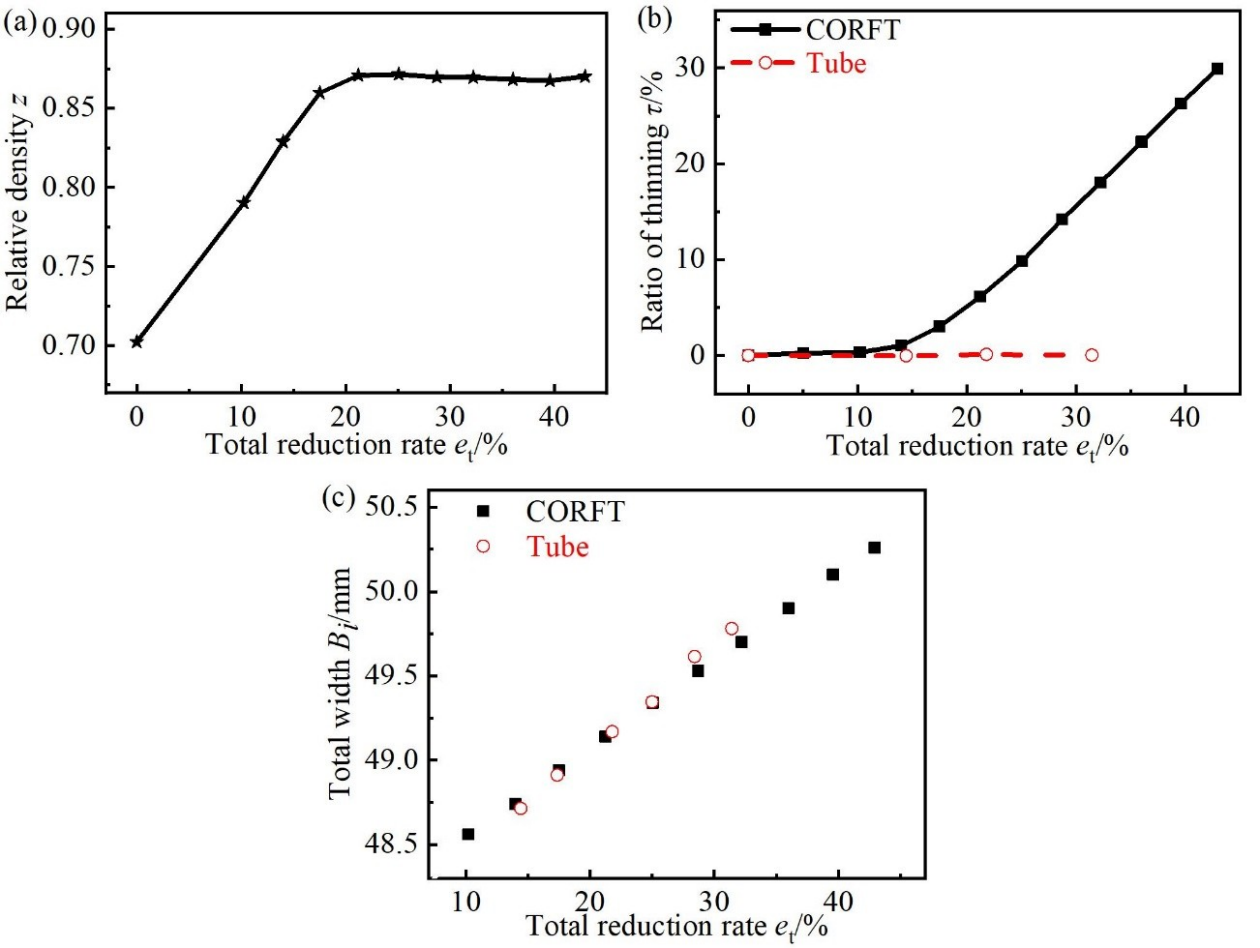
Parameters deduced by the flat rolling experiments. (a) Relative density of the CORFT, (b) ratio of thinning for the CORFT and the tube, and (c) total width of the CORFT and the tube.

To study the deformation laws for multiple passes in the flat rolling process of the CORFT and the tube, trend charts between the relative density *z* of the core material and the total reduction rate *e*_t_ were drawn, as shown in Fig 8(A). Trend charts between the ratio of thinning *τ* and the total width *B* of the rolled billets and the *e*_t_ were drawn, as shown in Fig 8(B) and 8(C).

As shown in Fig 8(A), with the increase in *e*_t_, the relative density *z* of the core material increased and then remained unchanged. When *e*_t_ was less than or equaled to *e*_c_, the *s* remained unchanged. Thus, with the increase in *e*_t_, the inner area of the steel tube of the CORFT decreased. Then *z* and *p*_ix_ increased. the Trend for the ratio of thinning and elongation increased and then the growth rate of *z* decreased until it approached zero. When the growth rate of *z* was zero and *e*_t_ increased, the *z* remained unchanged, that is, *z* had an ultimate value of *z*_U_, which was 0.868.

As shown in Fig 8(B), with increasing *e*_t_, the ratio of thinning *τ* for the tube was always zero. The value of *τ* for the CORFT was zero at first, and when *e*_t_ became larger than *e*_*c*_, *τ* started to increase, and the growth speed increased first and then remained unchanged. For the flat rolling process of the tube, only the outer steel tube bent, and the wall thickness remained unchanged because the inner surface of the steel tube was a free surface. For the flat rolling process of the CORFT at the initial stage of deformation, *p*_ix_ increased with increasing *e*_t_, but *p*_ix_ was not sufficient to facilitate the thinning of the outer steel tube; thus, the value of *τ* was always approximately zero. When *e*_t_ became larger than *e*_c_, *p*_ix_ became sufficient, and *τ* began to increase. Meanwhile, the growth speed of *τ* increased first and then remained unchanged because *z* increased first and then remained unchanged; that is, *p*_ix_ followed the same change law.

As shown in Fig 8(C), the total width *B* of the CORFT and tube both increased with increasing *e*_t_, and their curves were basically in coincidence. For the flat rolling process of the CORFT and tube, with increasing *e*_t_, *B* increased because fresh metal turns over to the roll surface continuously.

### 4.2 Verification of several hypotheses

The line segment thickness (the assumed thickness adopted in the uniform thickness hypothesis) *s*, the length of neutral line *l*_neu_ and section area *F*_sec_ for different rolling process of the CORFT (shown in Fig 7(B)) were measured. Then, the average thickness $\bar{s}$ was calculated as follows;

$$\bar{s}=\frac{F_{\sec}}{l_{\mathrm{neu}}}$$ (54)

**Fig 9:**
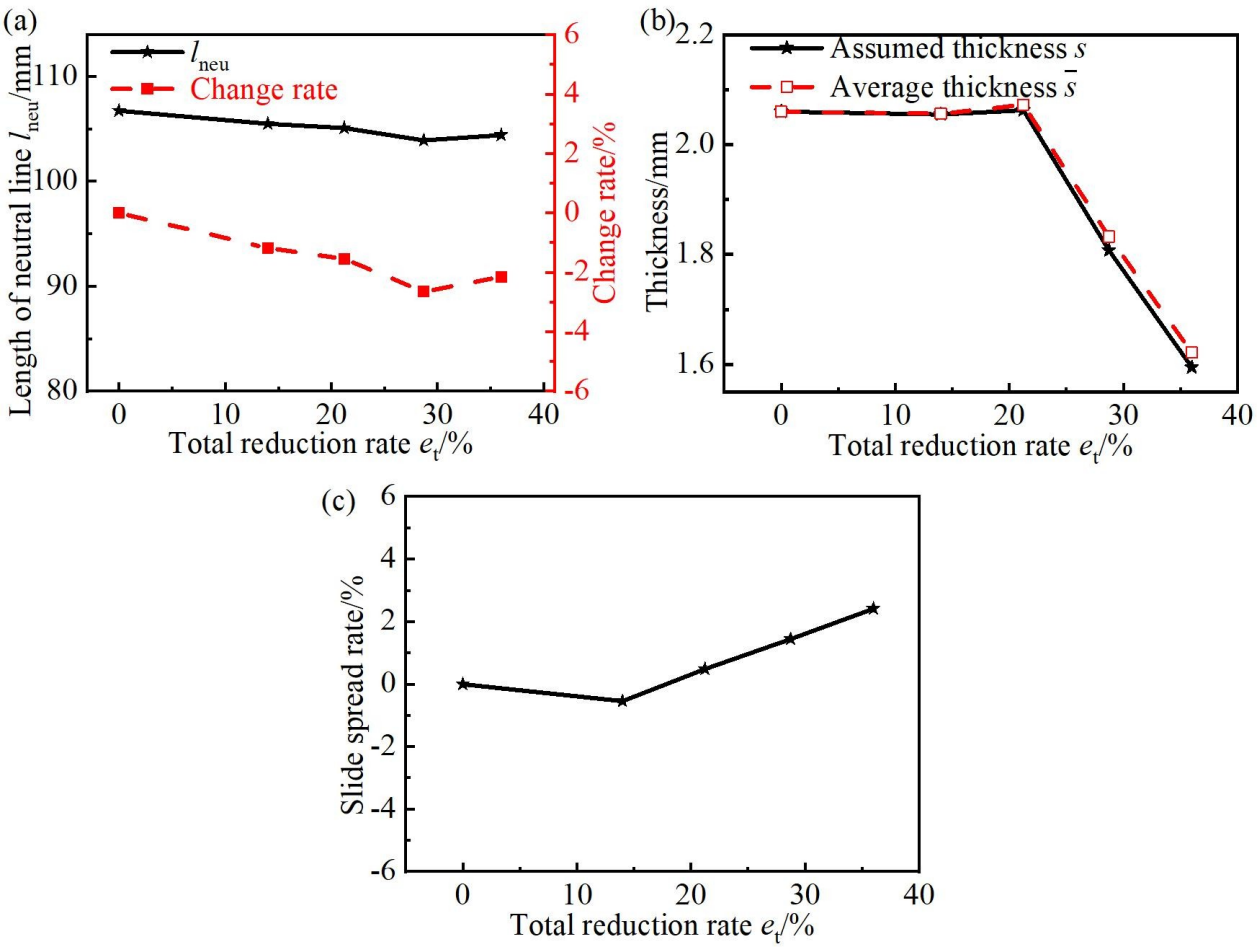
Validation of hypotheses on thickness, neutral line and slide spread rate. (a) Length of the neutral line, (b) comparison of the assumed thickness and average thickness, and (c) slide spread rate.

The trend diagrams of the thickness, the length of the neutral line and the slide spread rate in the different total reduction rate *e*_t_ are given in Fig 9. As shown in Fig 9(A), with the increase in the reduction rate *e*_t_, the length of the neutral line *l*_neu_ changed little; the change rate was within the range of -2.65% to 0%. Fig 9(A) verified the feasibility of the hypothesis regarding neglecting the change in *l*_neu_ during the rolling process of the CORFT. As shown in Fig 9(B), with the increase in the total reduction rate *e*_t_, the difference between the assumed thickness *s* and the average thickness $\bar{s}$ was very small, the error rate was within the range of -1.66% to 0%. Fig 9(B) verified the feasibility of the uniform thickness hypothesis (the thickness of the line segment was equal to the bending segment). As shown in Fig 9(C), with the increase in the total reduction rate *e*_t_, the slide spread rate was always very small; the value was within the range of -0.5% to 2.4%. Fig 9(C) verified the feasibility of the hypothesis regarding neglecting the slide spread rate during the rolling process of the CORFT.

### 4.3 Precision analysis of theoretical models

**Fig 10:**
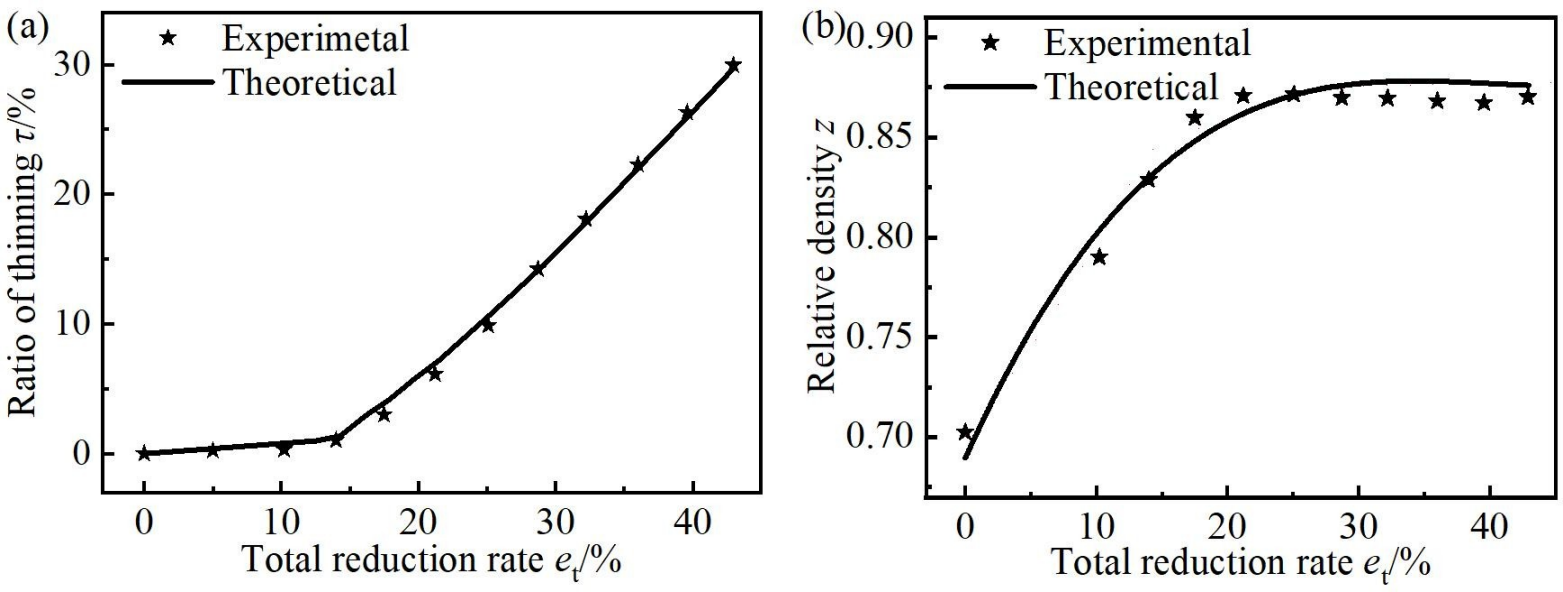
Comparison of the analytical and experimental results of the CORFT. (a) Ratio of thinning, and (b) relative density.

For the flat rolling process of the CORFT, the charts for the curves between *τ* and *e*_t_ are shown in Fig 10(A). The above two charts show that, with increasing *e*_t_, both the theoretical and the experimental values of *τ* were approximately zero at first. When *e*_t_ became larger than *e*_c_, *τ* began to increase, and the growth speed increased first and then remained unchanged. The error value between the two *τ* values was within the range of -0.27% to 1.14%. For the flat rolling process of the CORFTs, the charts for curves between the *z* and the *e*_t_ are shown in Fig 10(B). The above two charts show that, with increasing *e*_t_, both the theoretical and the experimental values of *z* increased first and then remained unchanged. The error ratio between the two *z* values was within the range of -1.05% to 0.99%. In addition, the theoretical and the experimental *z*_U_ values were 0.876 and 0.868, respectively. The error ratio between the two *z*_U_ values was within 1.0%. Fig 10 validated the accuracy of predicting *τ*, *z* and *z*_U_ using the theoretical equations.

### 4.4 Effect of rolling parameters on roll force

**Fig 11:**
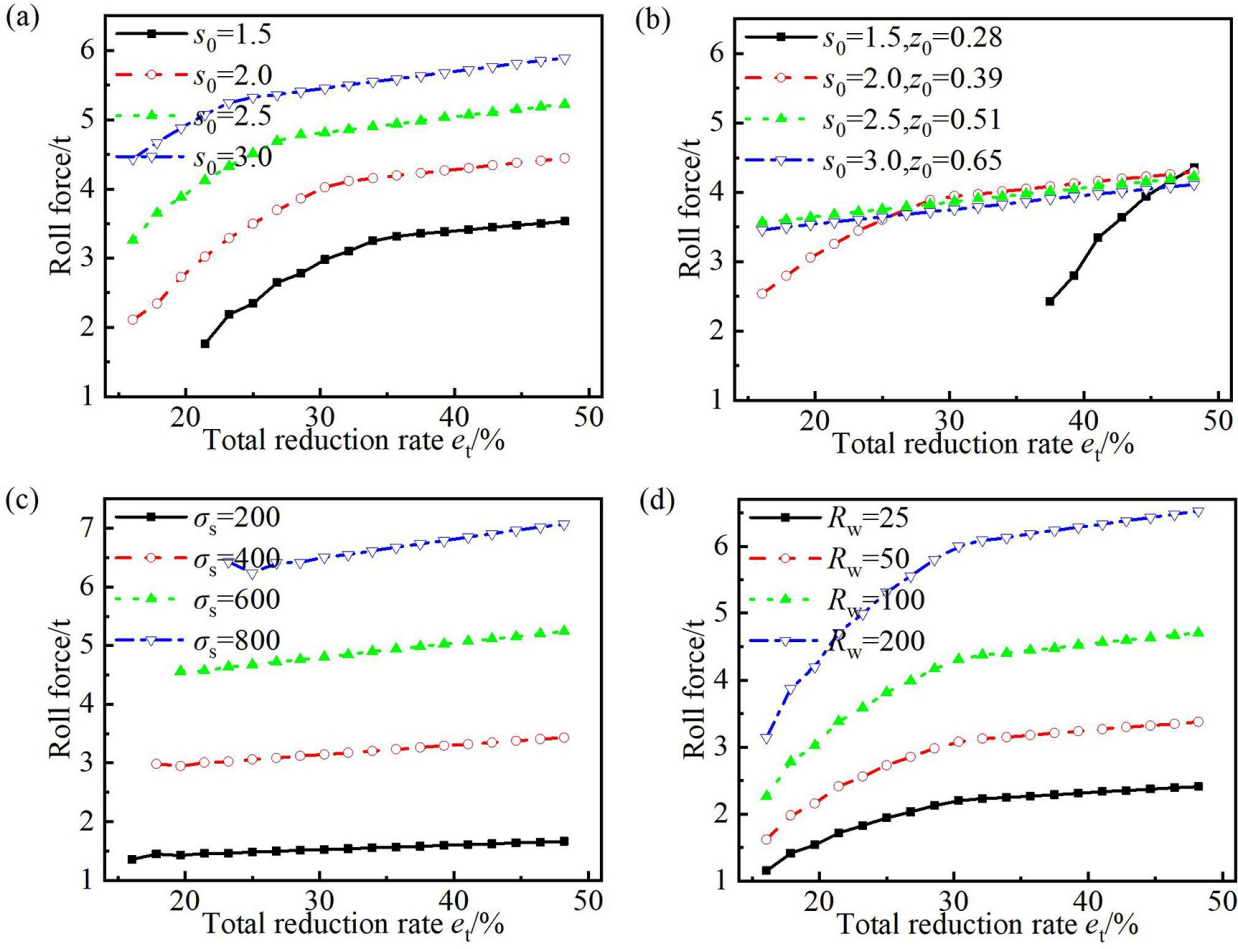
Effect of parameters on roll force. (a) Filling wall thickness, (b) filling wall thickness with the same mass ratio between the core material and the steel tube in a section, (c) yield strength of the steel tube, and (d) work roll radius.

During the rolling process of CORFTs, the roll force was an important parameter. It was related to the selection of rolling mills and the energy consumption. The curves between the roll force and total reduction rate *e*_t_ are drawn in Fig 11. The curves demonstrate that the roll force increased with increasing *e*_t_, and the increasing speed decreased first and then stabilized at a small value.

Fig 11(A) shows that the roll force increased with the increasing initial wall thickness *s*_0_. With the increase in the parameter *s*_0_, the area ratio *φ* between the core material and the steel tube in a section decreased and the thickness reduction of the outer steel tube increased. Thus, the roll force increased with increasing *s*_0_.

Fig 11(B) shows that at the same mass ratio *Φ*, the roll force increased with increasing *z*_0_ when the *e*_t_ was small, and remained unchanged with the change of *z*_0_ when *e*_t_ was large. When *e*_t_ was small, the volumetric shrinkage of the core material was large, and the reduction of wall thickness for the outer steel tube increased with increasing *z*_0_; thus, the roll force increased. When *e*_t_ was large, the volumetric shrinkage was small, the relative density of the core material approached one value (*z*_U_), and the area ratio *φ* remained unchanged with increasing *z*_0_; thus, the roll force remained unchanged.

Fig 11(C) shows that the roll force increased with increasing *σ*_s_ of the steel tube. With the increase in *σ*_s_, the deformation resistance increased and then the roll force increased.

Fig 11(D) shows that the roll force increased with increasing work roll radius *R*_w_. With increasing *R*_w_, the length of the deformation zone increased and then the roll force increased.

**Fig 12:**
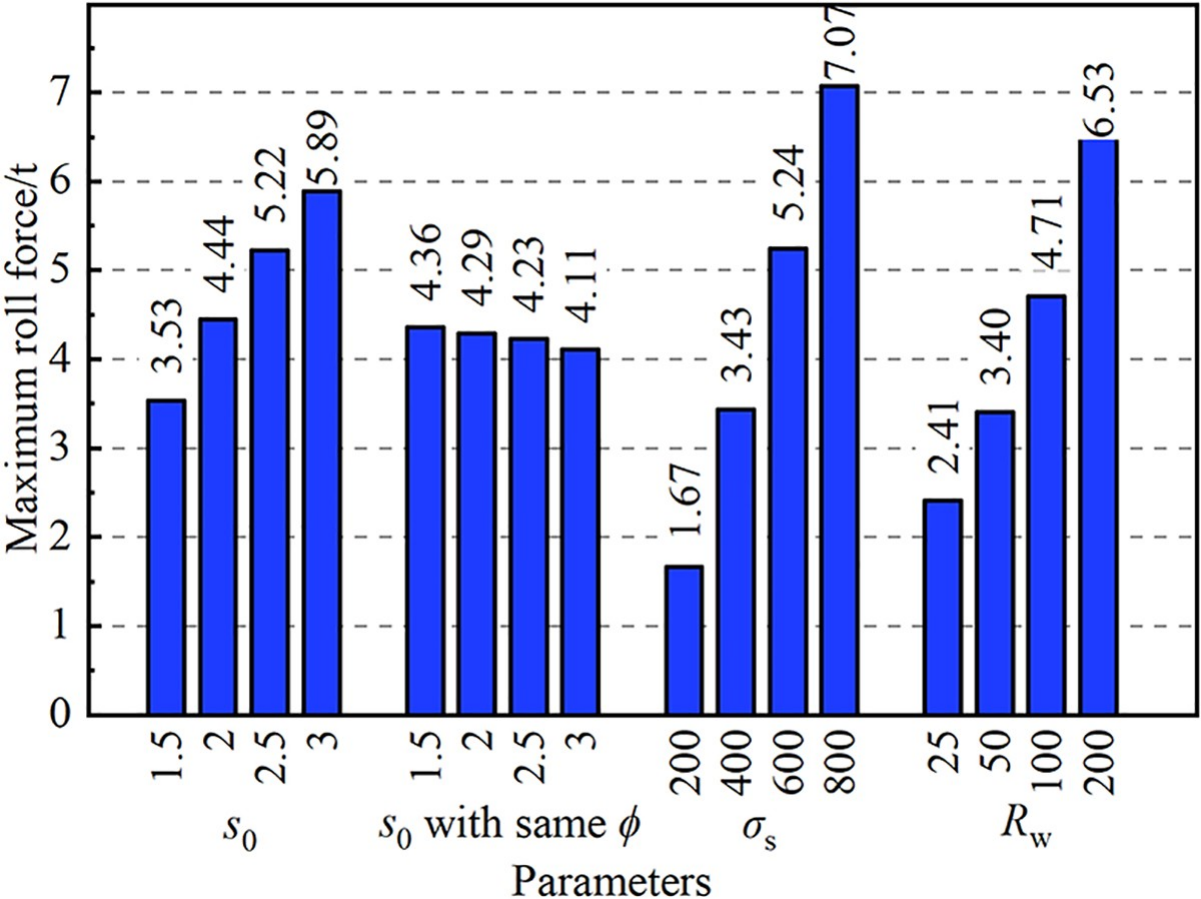
Effect of parameters on maximum roll force.

The maximum roll force is useful in mill design, and it needs to be quantitative analyzed, as shown in Fig 12. The above graph shows that the parameters *s*_0_, *σ*_s_ and *R*_w_ had obvious influences on the maximum roll force, but *s*_0_ had little influence on the maximum roll force when *Φ* remained unchanged. When *z*_0_ remained unchanged and *s*_0_ increased from 1.5 mm to 3.0 mm, the value of the maximum roll force increased from 3.53 t to 5.89 t with a changing rate of 66.9%. When *Φ* remained unchanged and *s*_0_ increased from 1.5 mm to 3.0 mm, the value of the maximum roll force was within the range of 4.11 t to 4.36 t. When *σ*_s_ increased from 200 MPa to 800 MPa, the value of the maximum roll force increased from 1.67 t to 7.07 t with a changing rate of 323.4%. When *R*_w_ increased from 25 mm to 200 mm, the value of the maximum roll force increased from 2.41 t to 6.53 t with a changing rate of 170.1%.

Overall, the limiting conditions for larger maximum roll force included a larger initial wall thickness, a higher yield strength of the steel tube, and a larger work roll radius.

### 4.5 Effect of rolling parameters on the ultimate value of the relative density

**Fig 13:**
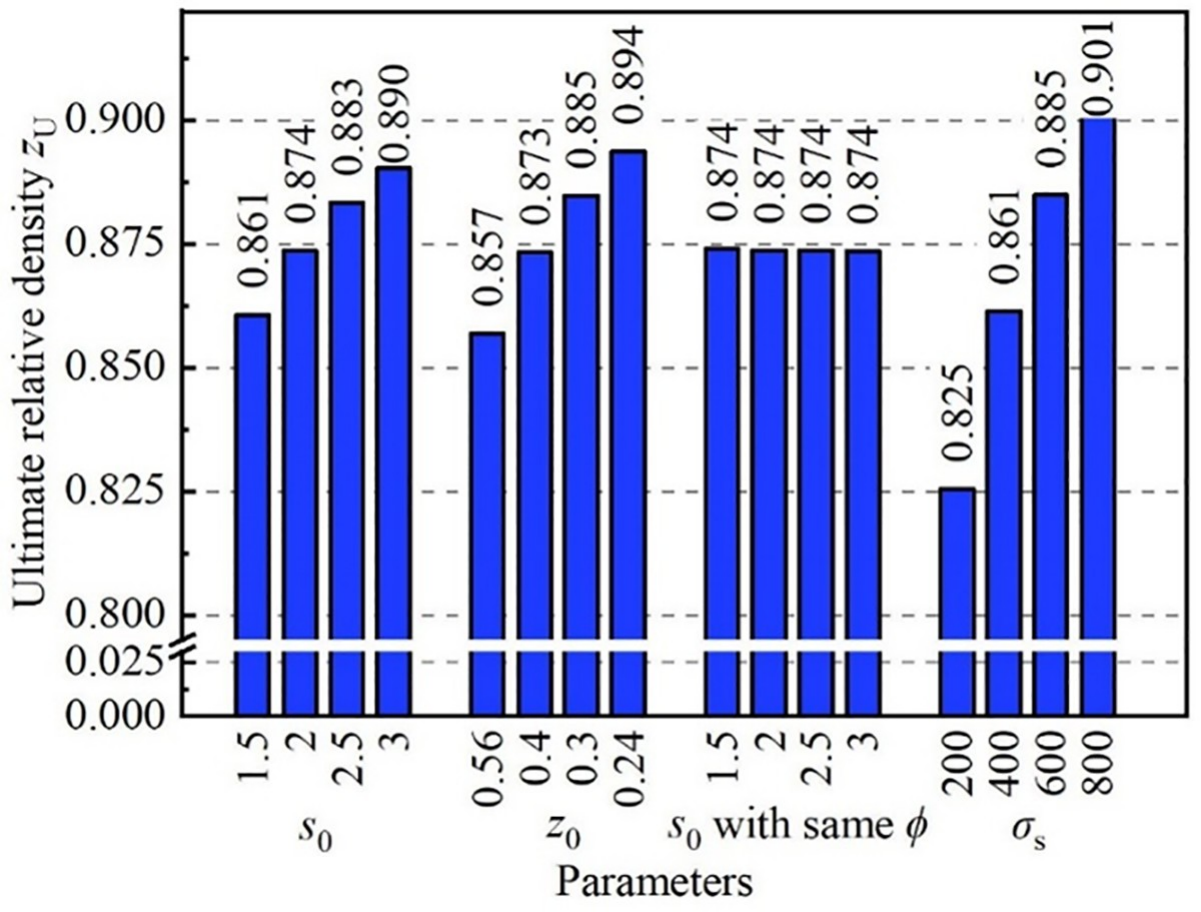
Effect of parameters on the ultimate value of the relative density.

*z*_U_ is an important parameter; with a larger *z*_U_, the holding force on the inner surface of the steel tube provided by the core material was larger, and the performance of flattening resistance for the CORFT was better. To study the influence of different rolling parameters on *z*_U_, a quantitative study on *z*_U_ was conducted, and the results are shown in Fig 13.

Fig 13 shows that the parameters *s*_0_, *z*_0_ and σ_s_ had obvious influences on *z*_U_, but *s*_0_ had little influence on *z*_U_ when *Φ* remained unchanged. When *z*_0_ remained unchanged and *s*_0_ increased from 1.5 mm to 3.0 mm, the value of *z*_U_ increased from 0.861 to 0.890 with a changing rate of 3.4%. When *s*_0_ remained unchanged and *z*_0_ decreased from 0.56 to 0.24, the value of *z*_U_ increased from 0.857 to 0.894 with a changing rate of 4.3%. When *Φ* remained unchanged and *s*_0_ increased from 1.5 mm to 3.0 mm, the value of *z*_U_ always equaled 0.874. When *σ*_s_ increased from 200 MPa to 800 MPa, the value of *z*_U_ increased from 0.825 to 0.901with a changing rate of 9.2%.

Overall, in order to obtain a CORFT with good flattening resistance performance, that is a CORFT with a high *z*_U_, rolling parameters with a small *Φ* and a large *σ*_s_ were adopted. In order to obtain a small *Φ*, rolling parameters with a large *s*_0_ or a small *z*_0_ were adopted.

## 5 Conclusions

(1) Adopting the plan deformation condition, an analytical model based on the slab method was developed. This model contained equations to calculate wall thickness *s*, relative density *z* of the core material, and roll force *P*.

(2) Validation experiments of flat rolling were conducted, and workpieces of the CORFT were obtained. Parameters including the ratio of thinning and relative density were calculated and compared with the theoretical values. The error value relative to the experimental data was -0.27% to +1.14% for the ratio of thinning *τ* and was -1.05% to +0.99% for the relative density *z*. The accuracy of the equations can satisfy the precision requirements in engineering.

(3) The influences of rolling parameters on roll force were studied. The limiting condition for larger maximum roll force included: larger initial wall thickness, higher yield strength of the steel tube, and larger work roll radius. When *s*_0_ increased from 1.5 mm to 3.0 mm, *σ*_s_ increased from 200 MPa to 800 MPa, or work roll radius increased from 25 mm to 200 mm, the maximum roll force increased from 3.53 t to 5.89 t, from 1.67 t to 7.07 t, or from 2.41 t to 6.53 t, respectively.

(4) To improve the performance of bending resistance, flattening resistance and corrosion resistance for the CORFT, the influences of rolling parameters on *z*_U_ were studied, and the process with a larger *σ*_s_, smaller *z*_0_, and larger *s*_0_ was adopted.

## References

[1] He K, Wang L. A review of energy use and energy-efficient technologies for the iron and steel industry. Renewable and Sustainable Energy Reviews. 2017; 70: 1022–1039. 10.1016/j.rser.2016.12.007

[2] Liu XH, Gao HT, Zhang SL, Qi JL. A kind of Core Filled Tube and it's preparation method. CN; 2017-05-31.

[3] Liu XH, Gao HT, Peng LG, Dong XX. A kind of Core Filled Tube/Core Filled Steel Bar and it's preparation method. CN; 2014-08-27.

[4] Hideo H, Akiyasu Y, Hidenori H, Ma YW. Recent advances in iron-based superconductors toward applications. Materials Today. 2018; 21: 278–302. 10.1016/j.mattod.2017.09.006

[5] Liu Y, Wang X, Gao Y. Three-dimensional multifilament finite element models of Bi-2212 high-temperature superconducting round wire under axial load. Composite Structures. 2019; 211: 273–286. 10.1016/j.compstruct.2018.12.027

[6] Zhang S, Li C, Ma X, Feng J, Shao B, Liu X, et al Fabrication of Bi-2223 High Temperature Superconducting Tapes With Groove Rolling Process. Ieee Transactions on Applied Superconductivity. 2020; 30: 1–4. 10.1109/TASC.2020.2978784

[7] Huang H, Ma Y, Yao C, Zhu Y, Zhang X, Dong C, et al Influences of Tape Thickness on the Properties of Ag-Sheathed Sr 1-x K x Fe 2 As 2 Superconducting Tapes. Ieee Transactions on Applied Superconductivity. 2018; 28: 1–5. 10.1109/TASC.2017.2779751

[8] Liang X, Liu Y, Li H, Gan Z, Liu B, He Y. An investigation on microstructural and mechanical properties of powder metallurgical TiAl alloy during hot pack-rolling. Materials Science and Engineering: A. 2014; 619: 265–273. 10.1016/j.msea.2014.09.091

[9] Nagasekhar AV, Tick-Hon Y, Ramakanth KS. Mechanics of single pass equal channel angular extrusion of powder in tubes. Applied Physics A. 2006; 85: 185–194. 10.1007/s00339-006-3677-y

[10] Han Z, SkovHansen P, Freltoft T. The mechanical deformation of superconducting BiSrCaCuO/Ag composites. Superconductor Science & Technology. 1997; 10: 371–387. 10.1088/0953-2048/10/6/001

[11] Korzekwa DA, Bingert JF, Podtburg EJ, Miles P. Deformation processing of wires and tapes using the oxide-powder-in-tube method. Applied Superconductivity. 1994; 2: 261–270. 10.1016/0964-1807(94)90012-4

[12] Pandheeradi M, Vaze SP, Yuan DW, Kuhn HA. Modeling and experimental validation of superconductor tape rolling. Journal of Manufacturing Science and Engineering, Transactions of the ASME. 2001; 123: 665–673. 10.1115/1.1371929

[13] Lu Y. Optimization of Cross-sectional Shapes of the Bi-2223/Ag Wires before Flat Rolling. Chinese Journal of Mechanical Engineering. 2009; 22: 890 10.3901/CJME.2009.06.890

[14] Lu YJ. Deformation analysis for the superconductor-silver interface in the Bi-2223/Ag tape rolling process. Proceedings of the 2017 7th International Conference on Manufacturing Science and Engineering (Icmse 2017). 2017; 128: 233–240. 10.2991/icmse-17.2017.42

[15] Jiang ZY, Tieu AK. A 3-D finite element method analysis of cold rolling of thin strip with friction variation. Tribology International. 2004; 37: 185–191. 10.1016/S0301-679X(03)00049-5

[16] Cavaliere MA, Goldschmit MB, Dvorkin EN. Finite element analysis of steel rolling processes. Computers & Structures. 2001; 79: 2075–2089. 10.1016/S0045-7949(01)00055-4

[17] Qi J, Liu X, Gao H, Chen J, Hu X, Yan S. Experiment and analytical model based on slab method for drawing process of core filled tube. International Journal of Mechanical Sciences. 2020; 165: 105152 10.1016/j.ijmecsci.2019.105152

[18] Rowe GW. Elements of metalworking theory. 1979.

[19] Razani NA, Mollaei Dariani B, Soltanpour M. Analytical approach of asymmetrical thermomechanical rolling by slab method. The International Journal of Advanced Manufacturing Technology. 2018; 94: 175–189. 10.1007/s00170-017-0801-4

[20] Sun J, Peng Y, Dong Z, Du X. Study on asymmetrical deformation and curvature of heavy cylinder rolling. International Journal of Mechanical Sciences. 2017; 133: 720–727. 10.1016/j.ijmecsci.2017.09.025

[21] Wang HY, Li X, Sun J, Wang ZH, Zhao DW, Zhang DH. Analysis of sandwich rolling with two different thicknesses outer layers based on slab method. International Journal of Mechanical Sciences. 2016; 106: 194–208. 10.1016/j.ijmecsci.2015.12.021

